# Rapid long-lasting biochemical and radiological response to sorafenib in a case of advanced hepatocellular carcinoma

**DOI:** 10.3892/ol.2013.1131

**Published:** 2013-01-11

**Authors:** ASSUNTA MARIA TERESA GERARDI, LUCA PIO STOPPINO, ARCANGELO LISO, LUCA MACARINI, MATTEO LANDRISCINA

**Affiliations:** 1 Units of Clinical Oncology, University of Foggia, Foggia I-71100, Italy; 2 Radiology, University of Foggia, Foggia I-71100, Italy; 3 Hematology, Department of Medical and Surgical Sciences, University of Foggia, Foggia I-71100, Italy

**Keywords:** hepatocellular carcinoma, sorafenib, α-fetoprotein, tumor shrinkage, targeted therapy

## Abstract

The multikinase inhibitor sorafenib has demonstrated an overall survival benefit in phase III hepatocellular carcinoma (HCC) trials and has become the new standard of care for advanced stages of this disease. However, in clinical practice, the vast majority of patients obtain disease stabilization and occasionally tumor shrinkage. Furthermore, the appropriate timing of sorafenib therapy initiation, in order to maximize its clinical activity, remains under debate. We report a case of 4-year sorafenib treatment in a patient with an advanced hepatitis C virus (HCV)-related HCC with extensive infiltration of the inferior *vena cava*. Sorafenib treatment induced a rapid complete biochemical response and a long-term favorable outcome. Additionally, no major toxicities or detrimental effects on quality of life were observed. Thus, it is likely that a subgroup of human HCC may be highly sensitive to sorafenib; new molecular determinants are required to select those patients who may benefit from this therapy. Furthermore, a prompt initiation of treatment when the hepatic function is not compromised is a prerequisite for maximizing the clinical activity of sorafenib.

## Introduction

Sorafenib is an oral multikinase inhibitor that is clinically active in advanced hepatocellular carcinoma (HCC) and is the present standard of care in the advanced stage of this particular malignancy. The analysis of sorafenib activity in two phase III placebo-controlled clinical trials undertaken in patients with advanced HCC in Western countries (the Sharp trial) and in the Asia-Pacific area, demonstrated that sorafenib induces an overall survival benefit that typically involves disease stabilization, whereas objective responses are rare (1–2). Herein, we report a case of an advanced hepatitis C virus (HCV)-related HCC with extensive infiltration of the inferior *vena cava* (BCLC stage C), which demonstrated a rapid complete biochemical response and a long-term favorable outcome with prolonged sorafenib treatment. Written informed consent was obtained from the patient.

## Case report

**Figure 1:**
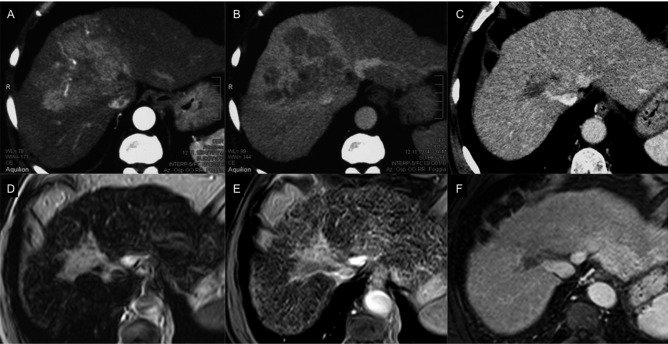
(A and B) Axial computed tomography (CT) scans at the baseline in arterial (A) and delayed (B) phases revealing a hypervascular mass in hepatic segment VIII. (C) Axial CT scan following 30 months of sorafenib therapy in the portal venous phase, revealing a hypodense lesion with a significant size reduction. (D and E) Double-contrast MRI scan following 35 months of sorafenib therapy, revealing a barely hyperintense lesion in a T2-weighted image after hepato-specific superparamagnetic iron oxide (SPIO) contrast agent administration (D) and a homogeneous lesion enhancement in a T1-weighted image after gadolinium administration (E), resembling fibrotic tissue. (F) MRI performed after 48 months of therapy, revealing substantial stability of the disease in a T1-weighted image following gadolinium administration.

In January 2007, a 72-year-old man with a clinical history of HCV-related hepatopathy was diagnosed with HCC. A computed tomography (CT) scan revealed a lesion of 1.5 cm in diameter in hepatic segment VIII with contrast enhancement in arterial phase (BCLC stage A, Child-Pugh A). Additionally, the patient’s α-fetoprotein (αFP) serum level was 487.3 ng/ml. Between March and November 2007, the patient was treated with 4 courses of percutaneous ethanol injections, which demonstrated an initial, but transitory, clinical benefit. In February 2008, the patient was referred to the Unit of Clinical Oncology, Department of Medical and Surgical Sciences (Foggia, Italy) due to rapid progression of the disease. The CT scan revealed a large mass (8.6 cm in diameter) in hepatic segment VIII, which was formed by multiple confluent nodules, infiltrating the inferior *vena cava*, altering the liver border and exhibiting typical arterial contrast enhancement (Figs. 1A and B). A chest CT scan did not detect extrahepatic metastasis. Based on these features, the malignancy was classified as BCLC stage C. The hepatic function was well-retained, with no clinical signs of liver dysfunction (Child-Pugh A) and an αFP serum level of 179.674 ng/ml.

As it was not possible to further use locoregional therapies, treatment with sorafenib (400 mg twice a day) was inititated. Notably, a rapid and sustained decrease in αFP serum level was observed with a normalization of the serum value (6.1 ng/ml) evident within 3 months of therapy. By contrast, the liver imaging showed an initial disease stabilization and, thereafter, a slow but progressive tumor shrinkage. Upon hepatic ultrasonography and CT scan the neoplastic lesion measured 7.4 cm in October 2008 (following 6 months of therapy), 5.3 cm in June 2009 and 4.0 cm in September 2010 (Fig. 1C). A double-contrast magnetic resonance imaging (MRI) scan with sequential use of hepato-specific superparamagnetic iron oxide (SPIO) and paramagnetic (gadolinium) contrast agents was performed in February 2011, revealing an area with slight signs of vascularization in hepatic segment VIII that resembled fibrotic tissue (Figs. 1D and E). Sorafenib was well-tolerated and the patient only presented with a grade 2 (NCI-CTCAE v3.0) hand-foot skin reaction, which was managed with urea cream; a grade 1–2 asthenia and occasional grade 2 hematological toxicities (leucopenia and thrombocytopenia), which were managed by 1–2 weeks of drug interruption. After 4 years, the patient has remained under sorafenib therapy, while αFP serum levels have remained within the normal range and radiological imaging has not exhibited any signs of disease progression (Fig. 1F).

## Discussion

Two issues are of particular significance in the present case report: i) the rapid biochemical complete response, followed by a slow but progressive and long-lasting tumor shrinkage; and ii) the lack of major toxicities, despite 4 years of essentially uninterrupted treatment. Sorafenib is the only molecular-targeted agent that has demonstrated a clinical benefit in phase III studies in advanced HCC (1–2). However, under sorafenib therapy, the majority of patients demonstrate disease stabilization, while tumor shrinkage is a less commom occurrence. Complete responses have not been noted in phase III clinical trials (1–2), while a number of recent studies have described major responses to sorafenib (3–7). Concordant with recent literature, the present case report suggests that a subset of HCCs may be particularly responsive to sorafenib and may undergo long-lasting tumor shrinkage. Thus, molecular determinants of susceptibility to sorafenib are required, in order to select patients that are more likely to benefit from this molecular-targeted agent and prolonged treatment. In this regard, we hypothesize that a rapid normalization of αFP serum levels may represent a surrogate marker of sensitivity to sorafenib. This hypothesis is concordant with recent reports demonstrating that the αFP response is a statistically significant prognostic factor for survival in HCC patients treated with sorafenib (8–9). Thus, early evaluation of αFP may be regarded as a reliable alternative to response evaluation criteria in solid tumors (RECIST) to capture sorafenib activity in HCC.

Notably, no major life-threatening toxicities or detrimental effects on quality of life were observed in the present case report, despite the 4 years of essentially uninterrupted treatment. This result is even more significant considering that the median duration of treatment in the phase III Sharp trial was 5.3 months (range 0.2–16.1) (1). A prompt initiation of sorafenib therapy, when the hepatic function has not yet been compromised, and an optimal management of side effects represent two prerequisites to allow a safe and, in the case of demonstrated efficacy, prolonged treatment for HCC patients. Consistently, recent studies have highlighted the importance of prolonged sorafenib administration, even at reduced doses, and personalized toxicity management to achieve tumor responses to sorafenib therapy (10).
